# Assessment of Damage to Nucleic Acids and Repair Machinery in *Salmonella typhimurium* Exposed to Chlorine

**DOI:** 10.1155/2009/201868

**Published:** 2009-07-19

**Authors:** M. H. Phe, M. Hajj Chehade, H. Guilloteau, C. Merlin, J. C. Block

**Affiliations:** Laboratoire de Chimie Physique et Microbiologie pour l'Environnement (LCPME), UMR 7564 CNRS, Nancy-Université, 405 rue de Vandoeuvre, 54600 Villers-lès-Nancy, France

## Abstract

Water disinfection is usually evaluated using mandatory methods based on cell culturability. However, such methods do not consider the potential of cells to recover, which should also be kept as low as possible. In this paper, we hypothesized that a successful disinfection is achieved only when the applied chlorine leads to both intracellular nucleic acid damage and strong alterations of the DNA repair machinery. Monitoring the SOS system responsiveness with a *umuC’-‘lacZ* reporter fusion, we found that the expression of this important cellular machinery was altered after the beginning of membrane permeabilization but prior to the total decline of both the cell culturability and the nucleic acid integrity as revealed by Sybr-II staining. Rapid measurement of such nucleic acid alterations by fluorochrome-based staining could be used as an alternative method for assessing the effectiveness of disinfection with chlorine.

## 1. Introduction

Chlorine (a mixture of HClO and ClO^−^) is the most widely used bactericidal agent for disinfection of drinking waters. Chlorine reacts with various biological molecules: proteins [1–3], lipids [4, 5], and nucleic acids [6–9]. By way of consequence, this strong oxidant affects structures and several metabolic processes such as membrane permeability [10–12], ATPase activity [13, 14], respiration [11], and the proton motive force of the cell [15]. All these deleterious effects were previously shown to occur very rapidly [16, 17].

One of the problems related to water disinfection with chlorine is linked to the control of the effectiveness of disinfection, which requires carrying out mandatory methods such as culturing bacteria on standard nutritive agar media. These mandatory methods give delayed results and, additionally, do underestimate the real number of viable bacteria in drinking water, especially when oxidative stress has been applied [11, 18, 19]. Then, the question of an optimal and effective dose of disinfectant (the dose which should prevent the repair of injured cells and their regrowth) has been left unanswered both (i) because the key functions or structures to be irreversibly targeted by the disinfection process have not been defined yet, and (ii) because there is no accurate and rapid method currently available for detecting irreversible injuries to be used as an indicator of treatment effectiveness.

Reactivity of HClO at lethal concentrations with nucleic acids is governed by chlorine diffusion into the cells and its direct action on cell polymers as well as by reactive oxygen species generated upon exposure to the oxidant [17, 20, 21]. Moreover, chlorine attacks preferentially exocyclic-NH2 groups of cytidine and adenosine at specific sites [8] and may also lead to DNA backbone cleavage [20, 22]. Saby et al. [23] first showed that chlorine-induced damage to nucleic acids could be revealed by the inability of fluorochromes, such as DAPI, to stain chlorinated bacteria. Other studies have corroborated this result and showed that chlorine reacting with nucleic acids in vitro and in vivo caused damage, thus resulting in a reduced fluorescence of the complex (nucleic acid + fluorochromes) stained with SYBR-II or propidium iodide (PI) [12, 24, 25].

A rapid analytical method which could confirm the irreversible and growth inhibitory nature of the damage suffered by chlorinated cells would clearly help practitioners to take the appropriate corrective actions to address various urgent needs (e.g., water disinfection, network cleaning, etc.). Therefore, there is a need for an alternative disinfection assessment method to be explored that would lie midway between usual methods such as culture, the limits of which are listed above, and methods measuring the complete ravages of cellular internal structure through observation of a fluorochrome staining drop.

In this paper, we hypothesized that a successful disinfection is achieved only when the applied chlorine concentration leads to both intracellular nucleic acid damage and strong alterations of the DNA repair machinery. Indeed, the inefficient fluorochrome staining of chlorinated bacteria could be used as a new criterion for a rapid water disinfection control. However, it does not give any indication on either the extent of the damage or its reversibility knowing that bacterial cells are equipped with a repair system. Therefore we investigated the effect of chlorine on the SOS system expression of *Salmonella typhimurium* (used as a laboratory model) and compared it to the loss of membrane permeability to propidium iodide (PI), DNA integrity assessed by Sybr-II staining, and bacterial culturability on nutritive agar medium. Finally, the pleiotropic effects of chlorine on various cell components are discussed, and we propose to rank these criteria for assessing disinfection efficiency and to define a threshold chlorine dose for a safe disinfection.

## 2. Materials and Methods

### 2.1. Bacterial Strain and Chlorine Treatment

All experiments were carried out using the strain TA1535/pSK1002 of *Salmonella typhimurium* [26], where the*umuC’-‘lacZ* fusion of plasmid pSK1002 can be used as a reporter to monitor the induction of the SOS system by genotoxic agents. *S. typhimurium* was grown in stirred batch culture at 37°C in trypticase soy agar (TSA) medium supplemented with 25 *μ*g mL^−1^ ampicillin, until OD_600_ reached 0.4. Bacterial cells were washed twice in PBS medium and adjusted to 2.8 × 10^8^ bacteria mL^−1^ in reverse osmosis water. Aliquots of the cell suspension were spiked with various concentrations of chlorine (commercial solution of bleach—Javel Jarrie water, Oxalis), ranging from 0.1 to 3 mg L^−1^ (measured as Cl_2_ by DPD method; Rodier [27]), and incubated for 90 minutes at 22 ± 1°C. A nonchlorinated control prepared in the same conditions was included in all assays. The pH of the assays ranged from 6.6 (nonchlorinated suspension) to 7.0 (chlorinated suspension with 3.5 mg Cl_2_ L^−1^). Although chlorine was very rapidly consumed in all assays (<2 minutes), all analyses were performed after any residual chlorine was neutralized by systematic addition of sodium thiosulfate.

### 2.2. Bacterial Counts

Bacterial cell culturability (Colony Forming Units, CFU) was estimated on TSA medium using plate count methods (incubation at 37°C for 72 hours), while total cell counts and membrane-altered cell counts were obtained by flow cytometry after staining, respectively, with Sybr-II and PI according to Phe et al. [12]. Additionally, the use of PI and Sybr-II fluorescent dyes allowed the assessment of nucleic acid integrity as previously shown by Phe et al. [12].

### 2.3. SOS/umu Chromotest Procedure

The bacterial response to DNA damage was assessed using the SOS reporter system of the strain by means of an “SOS *umu*-test” [26]. A 2 mL aliquot of the treated cells was buffered with 2 mL pH 7 PBS and was incubated at 37°C for 2 hours with 0.5 mL TSA medium to give the cells a chance to express the *umuC’-‘lacZ* fusion. The induction level of the SOS system was then evaluated by assaying *β*-galactosidase specific activity according to Miller [28]. The reactivity of the SOS system was controlled in duplicate experiments by adding a known genotoxic agent, the 4-nitroquinoline-1-oxide (4-NQO, final concentration 50 ng mL^−1^).

## 3. Results

### 3.1. Effect of Chlorine on Bacterial Counts and Fluorescence

**Figure 1:**
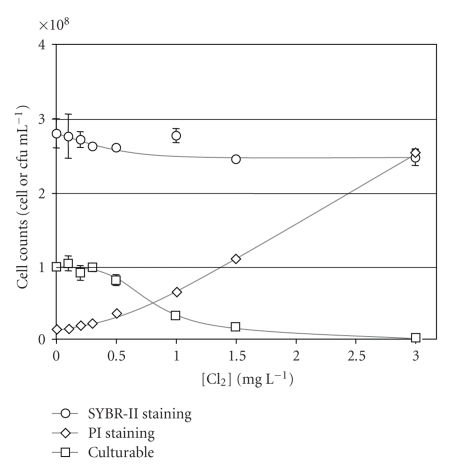
Numbers of CFU (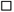), cells stained by Sybr-II (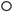), and cells stained by PI (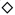) versus initial applied [Cl_2_] (mg L^−1^) (22 ± 1°C) of *Salmonella typhimurium*.

**Figure 2:**
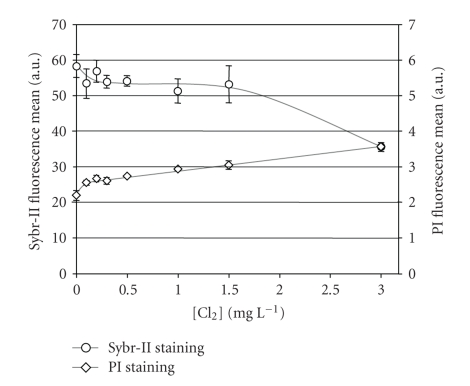
Mean fluorescence of *Salmonella typhimurium* cells stained either by SYBR-II (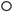) or by PI (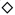) versus initial applied [Cl_2_] (mg L^−1^) (22 ± 1°C).

The total number of fluorescent cells counted by flow cytometry after cell staining with SYBR-II decreased by 12% for low chlorine exposure (0.3 mg Cl_2_ L^−1^) compared to the nonchlorinated control (Figure 1). This initial drop, which had been previously reported with chlorinated water samples [12, 25], could result from an alteration of a subset of fragile cells. For higher chlorine concentrations, the number of fluorescent cells remained steady but the fluorescence of the bacteria stained with SYBR-II decreased significantly after application of 1.5 mg Cl_2_ L^−1 ^(Figure 2).

Some cells were already detectable by PI staining before any chlorine treatment was applied (4% of the total cell counts), indicating the occurrence of chlorine-independent membrane alterations in this laboratory-grown suspension (Figure 1). The increase in PI-positive cells in the suspension was found to be chlorine concentration dependent. At 3 mg Cl_2_ L^−1^, virtually 100% of the cells were stained by PI (around 2.6 × 10^8^ PI+ cells mL^−1^) indicating membrane permeation of the major part of the bacterial population (Figure 1). However the increase in the number of PI+ cells was not proportionally correlated with the PI fluorescence increase (Figures 1  and 2), which could be explained by a partial alteration of the complex (PI + nucleic acid) formation in the *Salmonella* cells in agreement with the previous observations obtained with chlorinated *Escherichia coli* [25].

Without chlorine exposure, the culturable fraction of the *Salmonella* suspension represented no more than 35% of the total cell counts (Figure 1). The decrease in the number of colony forming units on TSA fits a simple inactivation kinetic model [29]. At 3 mg Cl_2_ L^−1^, 3.6 × 10^2^ bacteria mL^−1^ were still able to form colonies on plates even though these culturable bacteria may have been initially permeabilized by chlorine.

### 3.2. Effect of Chlorine on the Expression of the SOS System of *S. typhimurium*

**Figure 3:**
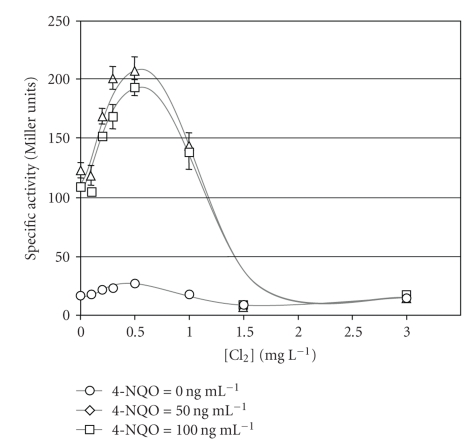
Specific activity of the *β*-galactosidase of *Salmonella typhimurium* exposed to chlorine or chlorine and 4-NQO.

As previously mentioned, the effect of chlorine on the SOS system was monitored using an *umuC’-‘lacZ* fusion. When bacteria were solely exposed to chlorine, the *β*-galactosidase specific activity increased only slightly (1.6-fold) between 0 and 0.5 mg Cl_2_ L^−1^ and went back to background level after treatment with 1.5 mg Cl_2_ L^−1^ and over (Figure 3).

When nonchlorinated bacterial suspensions were treated with 4-NQO, a genotoxic agent, the *β*-galactosidase specific activity was 6- and 7-fold higher compared to the control without 4-NQO. For bacterial suspensions subjected to chlorination followed by a 4-NQO treatment, the *β*-galactosidase specific activity increased from 100 to 200 Miller units, until [Cl_2_] = 0.5 mg L^−1^. This significant increase, compared to the assays without 4-NQO, showed that (i) the SOS system could still react after low chlorination (i.e., <0.5 mg L^−1^), and (ii) chlorine by itself had only a slight genotoxic effect. The synergistic effect occurring between chlorine and 4-NQO could be due to increased membrane permeability as demonstrated by PI fluorescence staining, leading in turn to better diffusion of the 4-NQO into the bacterial cells, and higher damage to the DNA.

From 0.5 to 1.5 mg Cl_2_ L^−1^, the decrease in *β*-galactosidase specific activity, from 200 to 15 Miller units, could result from general chlorine cytotoxicity against cell machineries including the SOS system. For higher chlorine concentrations, the SOS specific activity remained at background level indicating that most of the cells were not able to respond anymore.

## 4. Discussion

The pleiotropic effect of chlorine results from its reactivity with numerous biological molecules (on the cell surface and inside the cell after rapid diffusion), causing alterations of cell functions and inhibiting the bacterial culturability. In our assays carried out with laboratory-grown bacteria, we identified two subgroups in the initial cell population, one being more sensitive to the chlorine treatment than the other. This “sensitive” subpopulation was “bleached” (undetectable by Sybr-II) with 0.5 mg L^−1^ chlorine (Figure 1) while the remaining “resistant” subpopulation, representing about 88% of the initial cell population, persisted physically at chlorine concentrations as high as 3 mg L^−1^. The culturable counts started to decrease at chlorine concentrations for which only the resistant subpopulation persisted, thus suggesting that the sensitive subpopulation was already nonculturable prior to chlorine treatment. PI staining showed that before any chlorine treatment was applied, about 4% of the initial cell population displayed altered membrane properties. At this point, it is tempting to speculate that these 4% of membrane-damaged cells were part of cells forming the sensitive subpopulation.

The decrease observed in the mean fluorescence of Sybr-II-stained bacteria results from chlorine-damaged nucleic acids (especially at 3 mg Cl_2_ L^−1^) as expected from assays carried out with nucleic acid solutions [24], tap water bacteria, and *E. coli* suspensions [12, 25]. The increase in mean fluorescence after PI staining showed that chlorine affected membrane permeability, as reported by others [15], and allowed better diffusion of PI fluorochrome into the bacterial cells. It should be noted that, as evidenced by previous observations [25], the PI fluorescence plotted in Figure 2 probably should have been higher as chlorinated nucleic acids are not stained efficiently with propidium iodide.

As revealed by the specific activity of *β*-galactosidase, low chlorine exposure of cells had only a slight effect on the SOS system expression which is in agreement with reports from Le Curieux et al. [30], Thomas et al. [31], and Wlodkowski and Rosenkranz [32]. The SOS system's functionality was not altered for chlorine concentrations <0.5 mg L^−1^ as shown by its significant increase after addition of [4-NQO] = 50 ng mL^−1^ or 100 ng mL^−1^. The synergistic effect of chlorine combined with 4-NQO could be due to higher membrane permeability to 4-NQO as a result of chlorine treatment. However, for concentrations >0.5 mg Cl_2_ L^−1^, chlorine cytotoxicity overcomes rapidly the responses of the cells prohibiting any mutagenic effect measurement. Then chlorine potential mutagenic activity cannot be compared directly with that of less toxic or reactive agents [31]. The decrease in *β*-galactosidase specific activity for [Cl_2_] >0.5 mg L^−1^ can be the consequence of chlorine reacting with various cellular targets. Then, this loss in specific activity rather reflects the collapse of various cellular machineries, including the SOS system itself.

Interestingly, beyond the shift point of 0.5 mg L^−1^ of chlorine a substantial decrease both in culturability and SOS response was observed. This unexpected result sparks a renew interest in the culture method as chlorine-stressed nonculturable bacteria appear to be quite unable to repair damage caused by chlorine. However, at a chlorine concentration of 3 mg L^−1^, 3.6 × 10^2^ bacteria mL^−1^ were still culturable and SOS expression was not measurable anymore. Besides, only a partial reduction in the fluorescence of the bacterial population stained with SYBR-II was recorded for the same treatment (3 mg Cl_2_ L^−1^). This ranking of the responsiveness of the different methods may be caused by a relatively low sensitivity of the *umu*-test, especially with chlorinated bacteria, compared to that of the plate count method and by the partial alteration of nucleic acids by chlorine in the cells, which may be stained even for higher chlorine exposure. Nevertheless, whatever the mechanisms of chlorine action, it appears that this oxidant causes an immediate permeabilization of the cell envelopes combined with a loss in culturability and, at the very least for higher chlorine concentrations, a significant loss in fluorescence for the fluorochromes that stain nucleic acids.

## 5. Conclusions

This study has shown that shock chlorination on a relatively dense laboratory-grown bacterial population has pleiotropic effects on bacterial cells at the different levels of cellular organization. On the one hand, chlorine reacts at the bacterial cell surface increasing membrane permeability as revealed by a rise in the number of PI+ cells and in the mean fluorescence of PI-stained cells. On the other hand, chlorine diffuses into the cell and damages polymers, such as nucleic acids, as shown by a decrease in the mean fluorescence of Sybr-II-stained cells. Additionally, chlorine spoils the cellular machinery expression and de facto the SOS system expression. These new results support our initial hypothesis that efficient and safe disinfection (i.e., low risk of bacterial repair and regrowth) is definitively achieved only when a dramatic reduction in the fluorescence of DNA/RNA fluorochromes that stain bacterial cells is observed. Rapid measurement of such nucleic acid alterations by fluorochrome-based staining (results obtained within 1 hour) can be proposed as a new alternative method for assessing the effectiveness of disinfection.
